# ﻿Three new species of the genus *Alluaudomyia* Kieffer, 1913 (Diptera, Ceratopogonidae) from the National Park of Hainan Tropical Rainforest, China

**DOI:** 10.3897/zookeys.1112.83021

**Published:** 2022-07-14

**Authors:** Xiaoxiang Wu, Zehua He, Xiaodan Lin, Bin Deng, Qi Zhai, Jiahui Li

**Affiliations:** 1 Key Laboratory of Green Prevention and Control of Tropical Plant Diseases and Pests, Ministry of Education, College of Plant Protection, Hainan University, Haikou, 570228, China Hainan University Haikou China; 2 Hainan Yazhou Bay Seed Lab, Sanya, 572024, China Hainan Yazhou Bay Seed Lab Sanya China

**Keywords:** DNA barcodes, Hainan Island, *maculipennis* group, morphology, *parva* group

## Abstract

Three new species of the predaceous midges of genus *Alluaudomyia* Kieffer, 1913: *A.flavinotum* Wu & Li, **sp. nov.** of the *maculipennis* group, and *A.reflexuralis* Wu & Li, **sp. nov.** and *A.limu* Wu & Li, **sp. nov.** of the *parva* group, are described from the National Park of Hainan Tropical Rainforest, Hainan Island, China. Illustrations and COI barcodes (a fragment of the mitochondrial cytochrome c oxidase subunit 1) of the three new species are also provided. Associations of male and female specimens of two species (*A.reflexuralis* Wu & Li, **sp. nov.** and *A.limu* Wu & Li, **sp. nov.**) are supported by DNA barcodes. The *parva* group is reported from China for the first time.

## ﻿Introduction

The genus *Alluaudomyia* was erected in 1913 by Kieffer with the type species *Alluaudomyiaimparunguis* Kieffer, 1913. It is comprised of small but often strikingly marked predaceous midges (Wirth 1952; Glick and Mullen 1982; Spinelli and Wirth 1984; Sinha et al. 2005). The immature stages of *Alluaudomyia* species inhabit various aquatic habitats, such as ponds, lakes, bogs, fens, swamps, tree-holes, and margins of watercourses (Borkent 2014; Szadziewski et al. 2015). The larvae swim actively on the water surface film and feed on larvae of chironomids, mosquitoes, and ceratopogonids (De Meillon and Wirth 1991; Borkent and Spinelli 2007; Sarkar and Mazumdar 2019). Female adults are predators on adult Chironomidae (Downes 1978).

The genus consists of 203 extant species worldwide, making it one of the most diverse genera in the tribe Ceratopogonini (Borkent and Dominiak 2020; Borkent et al. 2022). Wirth and Delfinado (1964) recognized five species groups: *parva* group, *maculipennis* group, *marmorata* group, *xanthocoma* group, and *annulata* group. Yu et al. (2005) recognized another species group, the *desma* group, on the basis of Wirth and Delfinado’s (1964) classification. Thirty-three species of *Alluaudomyia* are known from China so far (Yu et al. 2005; Nie et al. 2009; Liu et al. 2011; Liu et al. 2021a, b), representing all species groups other than the *parva* and *annulata* groups. Six species have been recorded from Hainan Island, *A.columinis* Liu, Yan & Liu, 1996, *A.flexuosa* Yu & Hao, 2005, *A.formosana* Okada, 1942, *A.longzhouensis* Hao & Yu, 1991, *A.marginalis* Wirth & Delfinado, 1964, and *A.spinosipes* Tokunaga, 1962 (Liu et al. 1996; Yu et al. 2005; Wang 2018). Surveys of the ceratopogonid fauna of the National Park of Hainan Tropical Rainforest collected three species new to science. Comparative descriptions of these species are provided and supported by DNA barcodes.

## ﻿Materials and methods

### ﻿Specimens

Specimens were collected from Limushan Mountain and Bawangling Mountain in the National Park of Hainan Tropical Rainforest, Hainan Province, China, on 19–21 November 2020 and 21–23 May 2021. Seven to ten battery-powered UV light traps were set inside the forest, along the mountain road or streams and near small ponds each night (5 pm to 8 am next day). The traps were connected to sucking fans and collecting bottles filled with 75% ethanol. DNA was extracted for all type material in this study by non-destructive tissue digestion and cleared specimens were subsequently mounted onto microscope slides following the steps of Bellis et al. (2013). All specimens examined were mounted on slides and deposited in the Insect Collection of the College of Plant Protection, Hainan University, Haikou, China (**ICHU**).

### ﻿Morphology study

Images of the habitus of specimens kept in ethanol were taken before slide mounting using a camera DP72 attached to an Olympus SZX16 stereomicroscope. Images and measurements of specimens on slides were taken using a camera (P/N: YH5001) attached to a ShangGuang XSP-12CA microscope. Electronic drawings of male genitalia were made from photographs using Adobe Illustrator CC 2018 and Photoshop CC 2018. The geographical distribution was mapped by ArcMap 10.2 (Rinner and Voss 2013). The morphology terms and abbreviations used in the descriptions follow Wirth and Grogan (1988) and Szadziewski et al. (2015), with modifications of certain veins and cells as proposed by Borkent (2017). Measurements of series with values are given as “minimum value-maximum value (mean, *n* = number of measurements)”.

### ﻿DNA barcoding

DNA barcodes of the mitochondrial cytochrome c oxidase subunit 1 (COI) of the three new species were amplified and sequenced using standard protocols and primers (deWaard et al. 2008). New sequences were deposited in BOLD (http://www.boldsystems.org/index.php) and GenBank (accession numbers OM722201–OM722222). Combined COI sequences of the other two *Alluaudomyia* species published by others (*A.parva*, GenBank accession numbers JN291539, KM901343, KM920687, KR663659, KR953594, KR955895, KR957136, MG171582, MG180240; *A.quadripunctata*, GenBank accession number KT278187), phylogenetic analysis was performed using the neighbor-joining (NJ) method with *Stilobezziaantennalis* (Coquillett, 1901) (GenBank accession number MG175492.1) and *Stilobezziadiversa* (Coquillett, 1901) (GenBank accession number KM992971.1) as outgroups. An NJ tree was inferred by MEGA 7.0.14 (Kumar et al. 2016) using the nucleotide substitution model of Kimura-2-Parameter (K2P), bootstrap support values from 1000 replications. Intra- and interspecific genetic distances also were analyzed by MEGA software.

## ﻿Taxonomy

### ﻿Genus *Alluaudomyia* Kieffer, 1913

#### ﻿ Alluaudomyia flavinotum

Taxon classificationAnimaliaDipteraCeratopogonidae

Wu & Li sp. nov.

9C901EFF-B2A7-5794-96C3-2C845F8D66C1

https://zoobank.org/BF04B407-4E5C-46DA-9A63-DE834F7035A2

Figs 1 , 2

##### Type materials.

***Holotype*.** China • Hainan Island: ♀, Qiongzhong County, Limushan Town, Limushan National Forest Park: nearby stream, 186 m southeast Leige homestay, alt. 647 m, 19°10.50'N, 109°44.57'E, 19.XI.2020, Xiaoxiang Wu, Bin Deng & Zehua He leg., by light trap, cer1055.

***Paratypes*** (2♀). China • Hainan Island: 1♀, same data as holotype; cer1055-1 • 1♀, Limushan National Forest Park: nearby small hydropower station on Limuling Mountain, alt. 666 m, 19°10.46'N, 109°44.58'E, 19.XI.2020, Xiaoxiang Wu, Bin Deng & Zehua He leg., by light trap, cer1056.

##### Diagnosis.

This species belongs to the *maculipennis* group based on the wing with dark spots proximad of r-m crossvein and at the apex of vein R_3_, with dark streaks at the distal portion of longitudinal veins instead of spots, and a single spermatheca without diverticulum. This species can easily be distinguished from all other *Alluaudomyia* species by the coloration of the scutum, which is yellow and yellowish, without dark pigmentation, except for a dark longitudinal stripe at the base. In addition, its wing’s color pattern, with dark streaks on the distal portion of longitudinal veins and with five dark spots all covering the veins but no spots in cells, is also very diagnostic.

##### Description.

**Figure 1.**
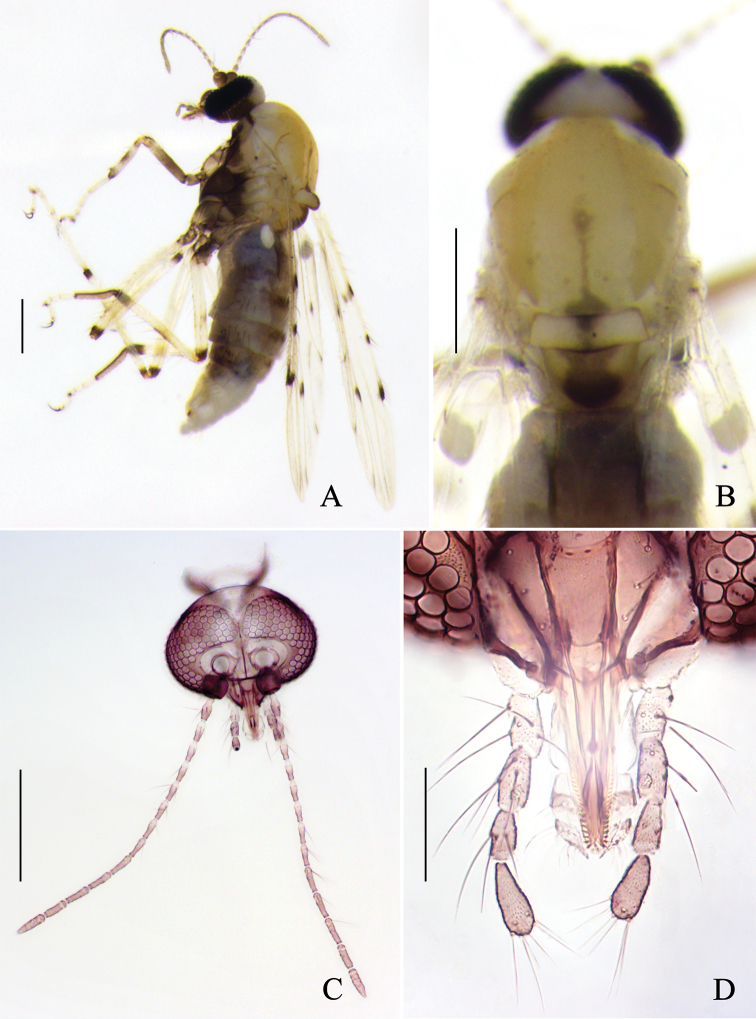
*Alluaudomyiaflavinotum* Wu & Li, sp. nov. **A** female, habitus in lateral view **B** thorax, dorsal view **C** head, dorsal view **D** maxillary palpi. Scale bars: 200 μm (**A, B**); 50 μm (**C, D**).

**Female.** Habitus (Fig. 1A) 1.28–1.42 mm (1.38, *n* = 3) in length.

***Head*** brown, except for the vertex yellowish-white, P/H 0.55–0.65 (0.61, *n* = 3). Eyes contiguous, bare. Antenna (Fig. 1C) brown with pedicel darker, flagellomeres 1–8 vasiform, pale at the base, distal 5 flagellomeres not considerably longer than basal flagellomeres, flagellum length 0.58–0.62 mm (0.60, *n* = 3), AR 0.74–0.81 (0.78, *n* = 3). Clypeus (Fig. 1D) brown with 3 pairs of setae. Palpus (Fig. 1D) brown with segments 1 and 2 slightly paler; third segment with a small, round, subapical pit, lengths 23–29 μm (26, *n* = 3), PR_III_ 1.61–1.85 (1.70, *n* = 3). Mandible with 13–15 teeth.

***Thorax*** generally yellow with dorsum lighter yellow, dark brown ventrally. Scutum (Fig. 1B) yellow except for anterolateral and central area light yellow, suture dark in basal half. Scutellum yellowish with dark medial marking, with 2 large setae. Postscutellum dark brown with anterolateral area yellowish.

**Figure 2.**
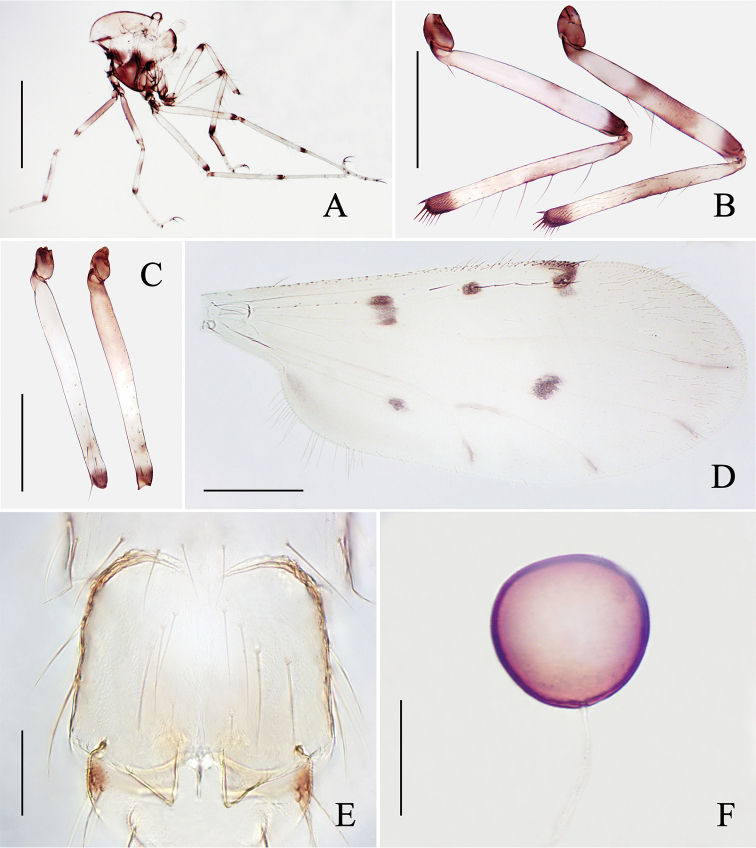
*Alluaudomyiaflavinotum* Wu & Li, sp. nov. **A** thorax, lateral view **B** hind legs **C** midfemora **D** wing **E** sternite 8 **F** spermatheca. Scale bars: 500 μm (**A**); 200 μm (**B–D**); 50 μm (**E, F**).

***Wings*** (Fig. 2D) pale with indistinct veins; five distinct dark spots cover the apex of cell r_2_, r-m crossvein, the midpoint of vein R_3_ and M_2_, and apical vein A; short dark streak covers veins M_1_, M_2_, CuA, and M_4_ subapically and base of vein M_4_; macrotrichia present along the radial vein, margin and apical 1/3 of the wing membrane; wing length 1.09–1.20 mm (1.13, *n* = 3), width 0.48–0.52 mm (0.49, *n* = 3), CR 0.64–0.66 (0.65, *n* = 3). Halter pale.

***Legs*** (Fig. 2A) bicolorous. Coxae and trochanters all brown; forefemur brown with a broad subapical pale ring, mid- and hind femur pale each with a dark apical ring; fore- and midtibiae dark brown at both ends, and broadly brown at middle in foretibia; hind tibia dark brown at distal end, and brown at middle; some specimens with an enlarged dark area at base of midfemur (Fig. 2C) and central area of hind femur and tibia (Fig. 2B); tarsi pale except for hind tarsomere 1 brown, fore- and midtarsomere 1 narrowly brown at the basal end. Hind tibial comb with 6–8 spines. Claws unequal for all legs, fore- and midclaws more slender. Tarsal ratio of foreleg TR_I_ 2.17–2.30 (2.23, *n* = 3), of midleg TR_II_ 2.97–3.13 (3.06, *n* = 3), of hind leg TR_III_ 2.96–3.16 (3.00, *n* = 3).

***Abdomen*** pale yellow. Sternite 8 (Fig. 2E) as long as broad, lateral margin strongly sclerotized, posterior margin separated medially forming two acute angles, with spike-like process at middle. Sternite 9 dark laterally. Single spermatheca (Fig. 2F) dark brown, round, measuring 67–76 μm (72, *n* = 3) by 66–75 μm (72, *n* = 3), neck absent.

**Male.** Unknown.

##### Etymology.

The name *flavinotum* refers to the yellow-colored scutum and scutellum.

##### Distribution.

China: Hainan Island: Qiongzhong County (Fig. 9).

##### Remarks.

The *maculipennis* group of China has been well studied, with 16 described species, and treated in the monographic work of Yu et al. (2005). Three new species were reported from China since that publication, including *A.haiyingi* Liu, Liu & Yu, 2011, *A.duchangensis* Liu & Yu, 2021, and *A.ruijinensis* Liu, Liu & Chen, 2021 (Liu et al. 2011, 2021a, 2021b). This new species runs to couplet 3 in the key to the *maculipennis* group by Yu et al. (2005). The new species resembles *A.lucania* Lee & Yu, 1997 in that couplet, differing in wing coloration pattern, which in *A.lucania* lacks a dark spot on the midpoint of R_3_, and with dark spots covering the longitudinal veins, with dark streaks in *A.flavinotum*. Additionally, the spermatheca of *A.lucania* has an obvious neck and the scutum is dark brown. The wing color pattern of *A.flavinotum* also looks similar to *A.typica* Chaudhuri, Das Gupta & Chaudhuri, 1972 from India, and *A.quinquepunctata* Tokunaga, 1940 from Japan. The new species can be distinguished with *A.typica* by the presence of a spot on the midpoint of vein R_3_ but with no dark spot in wing cell r_3_, and by the coloration of the scutum. It can be distinguished with *A.quinquepunctata* by the presence dark streaks covering on the distal portion of longitudinal veins.

#### ﻿ Alluaudomyia reflexuralis

Taxon classificationAnimaliaDipteraCeratopogonidae

Wu & Li sp. nov.

67136D96-C019-5818-9F21-6F2A350D7FE8

https://zoobank.org/05936EA5-EFC5-4D2A-89FC-BBA766B0F9C1

Figs 3 , 4 , 5

##### Type materials.

***Holotype*.** China • Hainan Island: ♂, Qiongzhong County, Limushan National Forest Park: a valley 500 m away from Limu Temple, alt. 585 m, 19°9.10'N, 109°45.31'E, 21.XI.2020, Xiaoxiang Wu, Bin Deng & Zehua He leg., by light trap, cer1089.

***Paratypes*** (4♂8♀). China • Hainan Island: 1♂, same data as the holotype, cer1089-1 • 1♀, Limushan National Forest Park: Management Committee East 610 m Mountain Rotten Wood, alt. 817 m, 19°10.61'N, 109°44.86'E, 19.XI.2020, Xiaoxiang Wu, Bin Deng & Zehua He leg., by light trap, cer1085 • 1♀, Village southeast 186 m by the stream, alt. 647 m, 19°10.46'N, 109°44.58'E, 19.XI.2020, Xiaoxiang Wu, Bin Deng & Zehua He leg., by light trap, cer1080 • 1♀, near mountain stream, 628 m east Limushan management building, alt. 817 m, 19°10.62'N, 109°44.87'E, 19.XI.2020, Xiaoxiang Wu, Bin Deng & Zehua He leg., by light trap, cer1081 • 1♀, nearby small hydropower station on Limuling Mountain, alt. 666 m, 19°10.46'N, 109°44.58'E, 19.XI.2020, Xiaoxiang Wu, Bin Deng & Zehua He leg., by light trap, cer1090 • 2♀, near a stream, 815 m northwest Xue’ershanfang hotel, alt. 686 m, 19°10.45'N, 109°43.95'E, 20.XI.2020, Xiaoxiang Wu, Bin Deng & Zehua He leg., by light trap, cer1082, cer1082-1 • 1♀, a valley, 5 km away from Limu Temple, alt. 582 m, 19°9.00'N, 109°45.20'E, 20.XI.2020, Xiaoxiang Wu, Bin Deng & Zehua He leg., by light trap, cer1079 • 1♀, near a valley, 500 m away from Limu Temple, alt. 585 m, 19°9.09'N, 109°45.31'E, 21.XI.2020, Xiaoxiang Wu, Bin Deng & Zehua He leg., by light trap, cer1084 • 3♂, near a valley, 4.2 km away from Limu Temple, alt. 567 m, 19°8.99'N, 109°45.22'E, 21.XI.2020, Xiaoxiang Wu, Bin Deng & Zehua He leg., by light trap, cer1100, cer1100-1, cer1100-2.

##### Diagnosis.

The new species belongs to the *parva* group based on its wing which has a single conspicuous dark spot at the apex of vein R_3_, two spermathecae without diverticula, and parameres with detached basal arms. The coloration of the wings and legs is quite similar to species of the *parva* group, but the males of this new species can easily be distinguished by the recurved gonostylus. The female of *A.reflexuralis* is very distinctive with the spermatheca having a rough surface near the neck, which is otherwise present only in *A.brevis* Wirth & Delfinado, 1964. These two species can be distinguished by four setae on the scutellum of *A.reflexuralis*.

##### Description.

**Figure 3.**
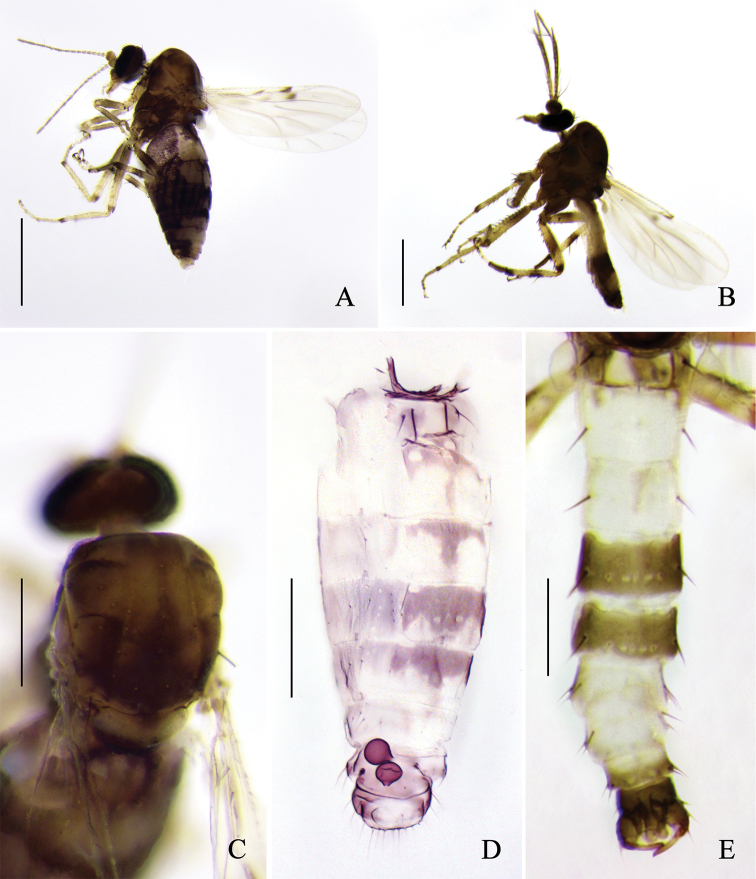
*Alluaudomyiareflexuralis* Wu & Li, sp. nov. **A** female, habitus in lateral view **B** male, habitus in lateral view **C** thorax of female, dorsal view **D** abdomen of female, dorsal view **E** abdomen of male, dorsal view. Scale bars: 500 μm (**A, B**); 200 μm (**C–E**).

**Female.** Habitus (Fig. 3A) 1.17–1.40 mm (1.38, *n* = 8) in length.

***Head*** brown, P/H 0.58–0.68 (0.65, *n* = 8). Eyes contiguous, bare. Antenna brown with darker pedicel, flagellomeres moderately long and tapering, flagellum length 0.51–0.59 mm (0.55, *n* = 8), AR 1.01–1.11 (1.07, *n* = 8). Clypeus brown with 4–6 setae. Palpus brown with segments 1–3 slightly paler; third palpal segment with a small, round sensory pit distally, length 31–37 μm (33, *n* = 8), PR_III_ 2.32–2.53 (2.40, *n* = 8). Mandible with 8–11 teeth.

***Thorax*** brown mottled dorsally, dark brown ventally. Scutum (Fig. 3C) dark brown with yellow anterolateral and central markings. Scutellum yellowish with dark marking at the middle, bearing 4 large setae. Postscutellum dark brown.

**Figure 4.**
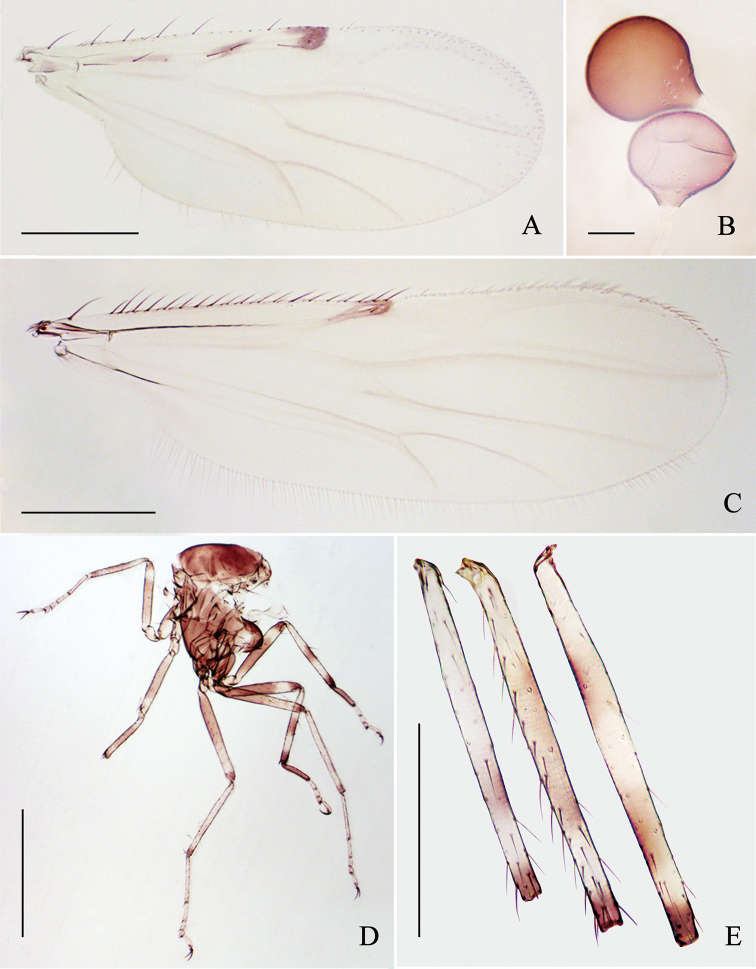
*Alluaudomyiareflexuralis* Wu & Li, sp. nov. **A** wing of female **B** spermathecae **C** wing of male **D** thorax of female, lateral view **E** mid tibia of female. Scale bars: 200 μm (**A, C, E**); 20 μm (**B**); 500 μm (**D**).

***Wings*** (Fig. 4A) with a conspicuous dark spot over the apex of vein R_3_ and a slim dark marking over vein R_1_; all veins slightly infuscated, excepting pale r-m crossvein, part of vein R, and vein R_3_; wing length 0.87–0.97 mm (0.90, *n* = 8), width 0.36–0.41 mm (0.39, *n* = 8), CR 0.52–0.56 (0.54, *n* = 8); macrotrichia rather sparse, present along the radial vein, margin and about apical 1/4 of wing membrane. Halter white.

***Legs*** (Fig. 4D) dark brown with pale rings. Coxae and trochanters all brown, of foreleg slightly paler; all femora brown, each with a subapical pale ring, hind femur pale at base; tibiae pale at basal and subapical part, broadly brown at middle, and narrowly dark brown apically, dark area of some specimens with an additional pale marking medially or the basal pale on midtibia (Fig. 4E); tarsi yellow or yellowish except for hind tarsomere 1 dark brown. Hind tibial comb with 6–7 spines; fore- and midclaws subequal, hind claws very unequal. Tarsal ratio of foreleg TR_I_ 1.81–2.00 (1.90, *n* = 8), of midleg TR_II_ 2.21–2.55 (2.35, *n* = 8), of hind leg TR_III_ 2.37–2.82 (2.63, *n* = 8).

**Figure 5.**
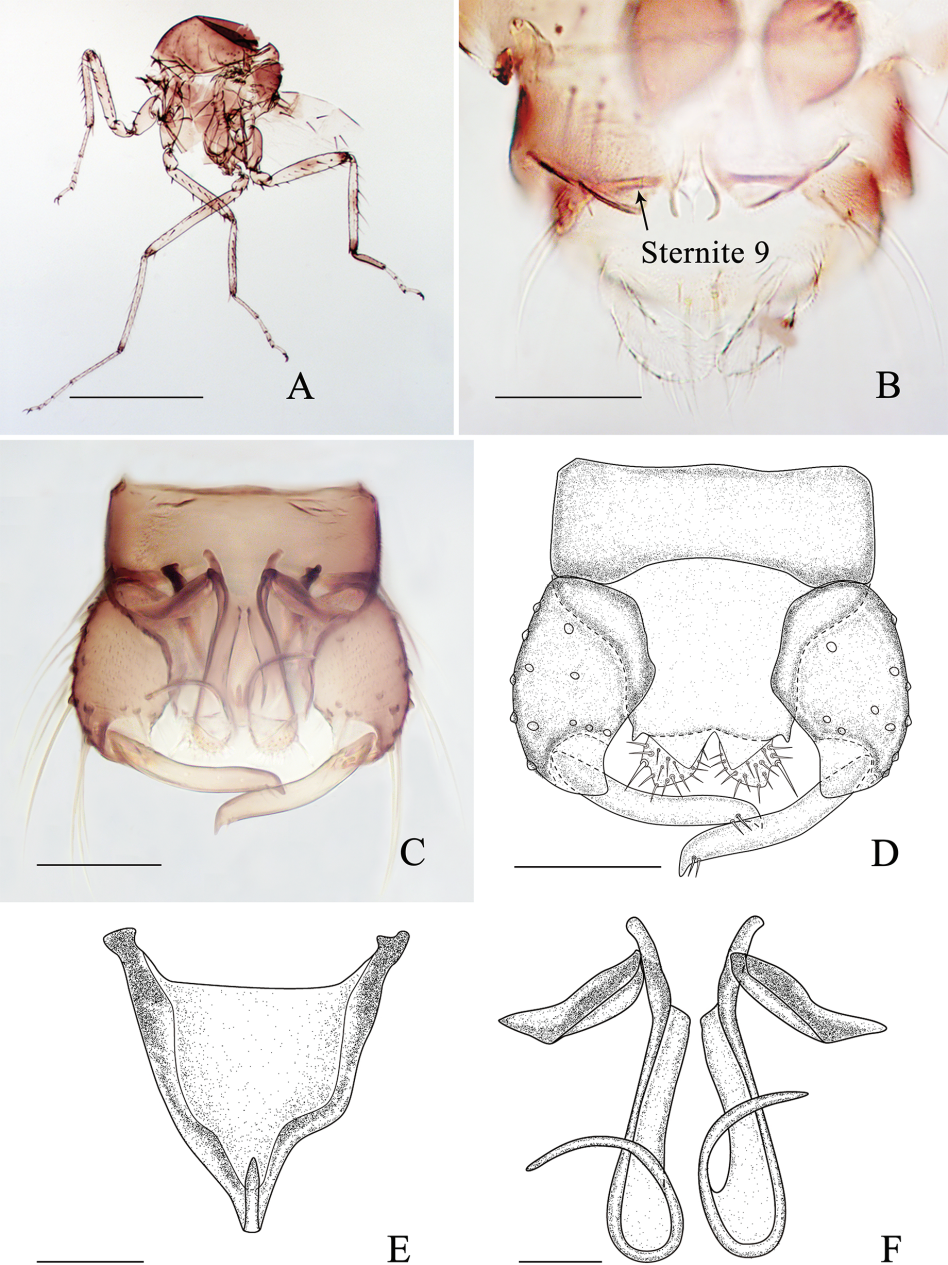
*Alluaudomyiareflexuralis* Wu & Li, sp. nov. **A** thorax of male, lateral view **B** sternites 8 and 9 of female, ventral view **C** male genitalia, ventral view **D** male genitalia with parameres and aedeagus removed, ventral view **E** aedeagus, ventral view **F** parameres, ventral view. Scale bars: 500 μm (**A**); 50 μm (**B–D**); 20 μm (**E, F**).

***Abdomen*** (Fig. 3D). Tergites 1–5 pale with brown markings. Sternite 8 moderately sclerotized, posterior margin with a pair of broad and short triangular processes. Sternite 9 brown, strongly narrowing from lateral side to the middle part (Fig. 5B). Two spermathecae (Fig. 4B) subequal, measuring 51–60 μm (55, *n* = 8) by 44–49 μm (47, *n* = 8) and 47–49 μm (48, *n* = 8) by 38–44 μm (41, *n* = 8), pear-shaped, with short, sclerotized neck, and surface with small hyaline markings near neck.

**Male.** Habitus (Fig. 3B) 1.33–1.70 mm (1.52, *n* = 5) in length. Generally similar to female, differing as follows:

***Antenna*** with pedicel dark brown, flagellomeres 1–10 pale brown, plumose hairs yellowish-brown, flagellomere 13 slightly shorter than flagellomere 12. Clypeus with 4 setae. Teeth absent. Wing (Fig. 4C) slim and slightly longer than in females, length 0.97–1.08 mm (0.94, *n* = 5), width 0.30–0.37 mm (0.33, *n* = 5), CR 0.46–0.48 (0.47, *n* = 5), and with a single conspicuous spot at the apex of cell r_2_. The banding pattern of legs (Fig. 5A) is similar to female but much lighter in color, all legs with two rows of spine-like setae. Abdomen (Fig. 3E) pale, tergites 4 and 5 with brown markings; tergites 1–5 with one long seta at each side, and tergites 6–8 with two.

***Male genitalia*** as Fig. 5C–F. Sternite 9 with very shallow caudomedial excavation. Tergite 9 short and broad, posterior margin almost truncated, with small apicolateral projections, cerci with scattered setae. Gonocoxite stout, bearing distinct short setae and sparse long setae; gonostylus slender, slightly recurved and pointed apically, surface smooth with sparse indistinct setae in line and two distinct subapical setae. Aedeagus (Fig. 5E) arched, basal arch low; distomedian process medium length, apex pointed, and strongly reflexed ventrally. Parameres (Fig. 5F) separate, each one with a detached basal arm broad, medial portion nearly straight, semi-embraced and tube-like, distal portion strongly curved, tapering, and pointed apically.

##### Etymology.

The name *reflexuralis* refers to the slightly recurved gonostylus; to be treated as an adjective.

##### Remarks.

Both the new species and the following described new species *A.limu* belong to the *parva* group, which is recorded from China for the first time. All specimens of *A.reflexuralis* were collected from the rainforest of Hainan Island from 567 to 817 m. The color patterns of the wings, legs of *A.reflexuralis* resemble that of *A.limu*, but the new species can easily be discriminated by the male and female genitalia. It is difficult to associate males with females for these two species just by morphological characters. DNA barcodes helped to correctly associate both sexes for these two species.

#### ﻿ Alluaudomyia limu

Taxon classificationAnimaliaDipteraCeratopogonidae

Wu & Li sp. nov.

3454B2EE-EEFF-5BE7-B79A-6456F79C7F14

https://zoobank.org/3757B74C-AFEE-456B-A704-BB08C2250AE2

Figs 6 , 7 , 8

##### Type materials.

***Holotype*.** China • Hainan Island: ♀, Qiongzhong County, Limushan Town, Limushan National Forest Park: near a stream, 815 m northwest Xue’ershanfang hotel, alt. 686 m, 19°10.45'N, 109°43.95'E, 20.XI.2020, Xiaoxiang Wu, Bin Deng & Zehua He leg., by light trap, cer1077.

***Paratypes*** (1♂4♀). China • Hainan Island: 2♀, Limushan National Forest Park: nearby stream, 186 m southeast Leige homestay, alt. 647 m, 19°10.50'N, 109°44.57'E, 20.XI.2020, Xiaoxiang Wu, Bin Deng & Zehua He leg., by light trap, cer1076, cer1076-1 • 1♂, near mountain stream, 628 m east Limushan management building, Limushan National Forest Park, alt. 817 m, 19°10.62'N, 109°44.87'E, 20.XI.2020, Xiaoxiang Wu, Bin Deng & Zehua He leg., by light trap, cer1097 [thorax missing] • 1♀, board road, 109 m south Badao, alt. 546 m, 19°4.99'N, 109°7.41'E, 21.V.2021; Xiaoxiang Wu, Bin Deng & Zehua He leg., by light trap, cer1131 • 1♀, Changjiang County, Qicha Town, Bawangling national natural reserve: Chicken coop near Yajia hotel, alt. 470 m, 19°5.10'N, 109°7.40'E, 21.V.2021; Xiaoxiang Wu, Bin Deng & Zehua He leg., by light trap, cer1138.

##### Diagnosis.

The new species belongs to the *parva* group, as proposed for *A.reflexuralis*. The color pattern of *A.limu* mostly resembles that of *A.reflexuralis* but can be distinguished by its straight and apical blunted gonostylus, interior forked distomedian process of aedeagus, and straight posterior margin of sternite 9 in males and the smooth surface of the spermatheca in females.

##### Description.

**Figure 6.**
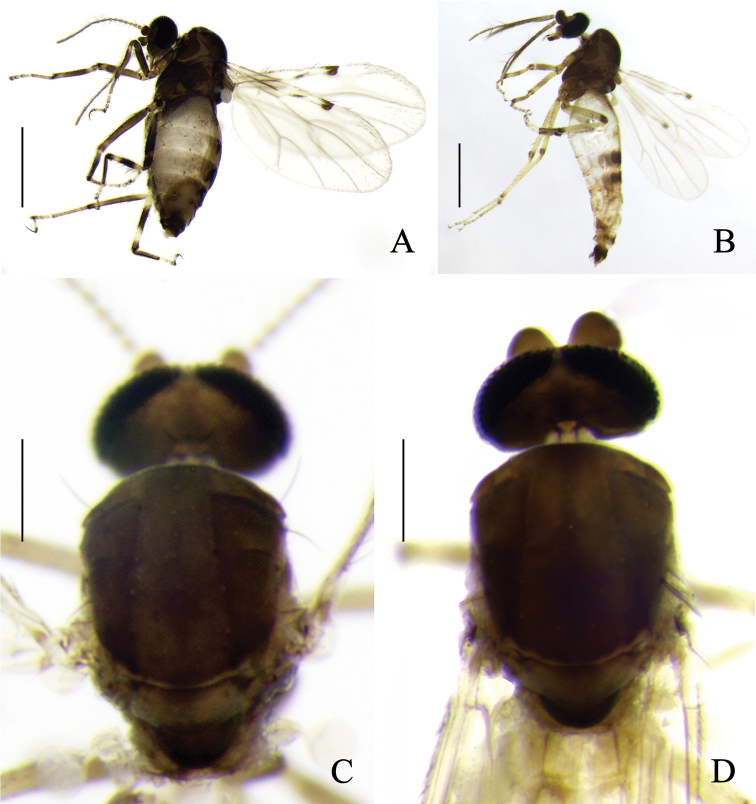
*Alluaudomyialimu* Wu & Li, sp. nov. **A** female, habitus in lateral view **B** male, habitus in lateral view **C** thorax of female, dorsal view **D** thorax of male, dorsal view. Scale bars: 500 μm (**A, B**); 200 μm (**C, D**).

**Female.** Habitus (Fig. 6A) 1.26–1.49 mm (1.36, *n* = 5) in length.

***Head*** brown, P/H 0.51–0.61 (0.57, *n* = 5). Eyes contiguous, bare. Antenna brownish with slightly darker pedicel, proximal flagellomeres elongate and tapering, flagellum length 0.58–0.69 mm, AR 1.02–1.10 (1.06, *n* = 5). Clypeus brown with 4–6 setae. Palpus brown with segments 1–3 paler; third palpal segment with a small round proximal sensory pit, length 31–37 μm (33, *n* = 8), PR_III_ 2.18–2.36 (2.28, *n* = 5). Mandible with 10–12 teeth.

***Thorax*** dark brown, yellowish brown laterally. Scutum dark brown with anterolateral areas and both sides of suture slightly paler (Fig. 6C). Scutellum yellowish, dark medially, with 4 setae. Postscutellum dark brown.

**Figure 7.**
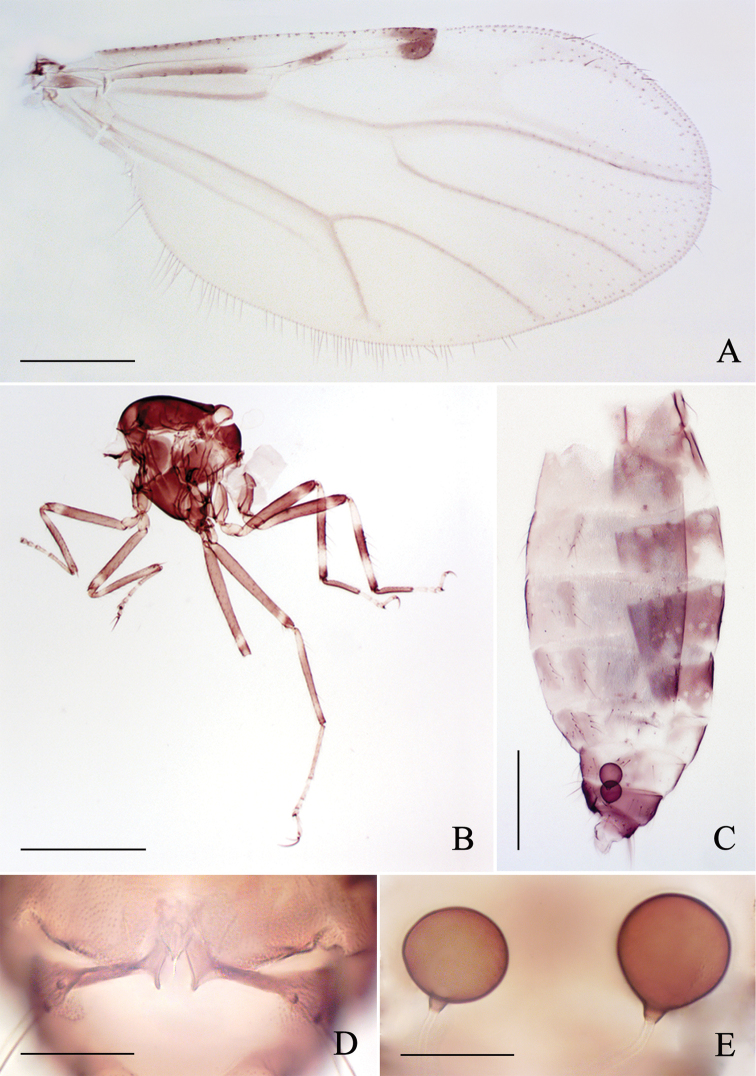
*Alluaudomyialimu* Wu & Li, sp. nov. **A** wing of female **B** thorax of female, lateral view **C** female abdomen, lateral view **D** sternite 9, ventral view **E** spermathecae. Scale bars: 200 μm (**A, C**); 500 μm (**B**); 50 μm (**D, E**).

***Wings*** (Fig. 7A) with a single conspicuous dark spot over apex of vein R_3_, vein infuscated except for r-m crossvein and part of veins R and R_3_ whitish; wing length 1.16–1.22 mm (1.07, *n* = 5), width 0.46–0.56 mm (0.53, *n* = 5), CR 0.52–0.56 (0.53, *n* = 5). Halter white.

***Legs*** (Fig. 7B) most in brown with pale rings. Coxae and trochanters all brown, of coxae slightly paler; fore- and midfemur brown, each with a subapical pale ring; hind femur pale at base and with a subapical pale ring; all tibiae brown with basal and subapical pale rings, and pale rings on hind tibia wider and more distinct; fore- and midtarsi pale brown, hind tarsomere 1 brown, other tarsomeres of hind tarsi yellowish. Hind tibial comb with 6–8 spines; claws unequal, fore- and midclaws much slender than hind claws. Tarsal ratio of foreleg TR_I_ 1.92–2.11 (2.01, *n* = 5), of midleg TR_II_ 2.24–2.38 (2.32, *n* = 5), of hind leg TR_III_ 2.58–2.72 (2.72, *n* = 5).

***Abdomen*** (Fig. 7C). Tergites with brownish markings. Sternite 9 (Fig. 7D) heavily sclerotized, divided medially, each piece narrowing to rod shape at middle, broad at both ends, with a distinct projection at the internal end. Two suborbicular spermathecae (Fig. 7E) unequal in size, measuring 50–62 μm (57, *n* = 5) by 44–54 μm (51, *n* = 5) and 43–52 μm (49, *n* = 5) by 41–48 μm (46, *n* = 5), with obvious neck.

**Male.** Habitus (Fig. 6B) 2.00 mm in length.

Generally similar to female, differing as follows:

***Antenna*** with pedicel dark brown, flagellomeres 1–10 pale brown, plumose hairs yellowish-brown, flagellomeres 11–13 brown. Teeth absent. Wing with a single spot covering apex of cell r_2_, with a few setae at margin. Scutum as in female but a shade darker (Fig. 6D). Legs (Fig. 6B) coloration similar to but much paler than females; foreleg more or less yellow-brown, with basal and subapical narrow pale rings of femur and tibia; mid- and hind leg pale with distal dark ends of femur and tibia. Abdomen pale, except tergites 4 and 5 with brown markings.

**Figure 8.**
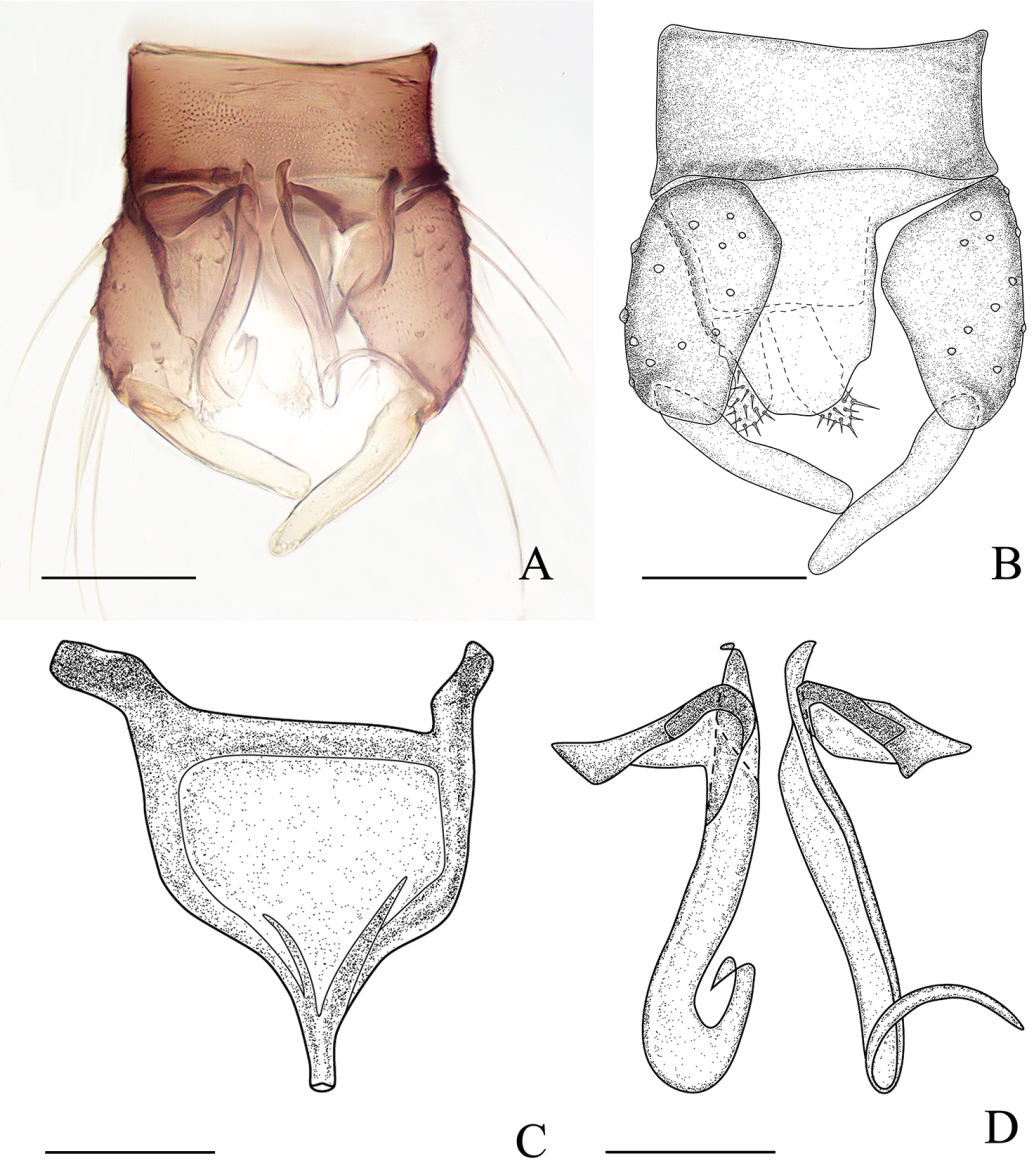
*Alluaudomyialimu* Wu & Li, sp. nov. **A** male genitalia, ventral view **B** male genitalia with parameres and aedeagus removed, ventral view **C** aedeagus, ventral view **D** parameres, ventral view. Scale bars: 50 μm (**A, B**); 20 μm (**C, D**).

***Male genitalia*** (Fig. 8A–D). Tergite 9 broad at the base, tapering apically but not pointed; cerci 1/2 as long as gonocoxite, oblong, setose. Sternite 9 moderately short, about 2 times longer than broad; both anterior and posterior margins almost straight. Gonocoxite stout with distinct short setae and sparse long setae. Gonostylus stout, nearly straight and blunt apically. Aedeagus (Fig. 8C) arched; basal arch low, with sclerotized anterior margin; two processes forked, protruding from base of distomedian process, tapering to pointed tip. Parameres (Fig. 8D) separate, each one with a broad, detached basal arm, medial portion semi-embraced, tube-like, distal portion strongly curved and tapering into a pointed apex.

##### Etymology.

The species is named for *Limu*, the mother lord of the Li ethnic minority on Hainan Island.

##### Distribution.

**Figure 9.**
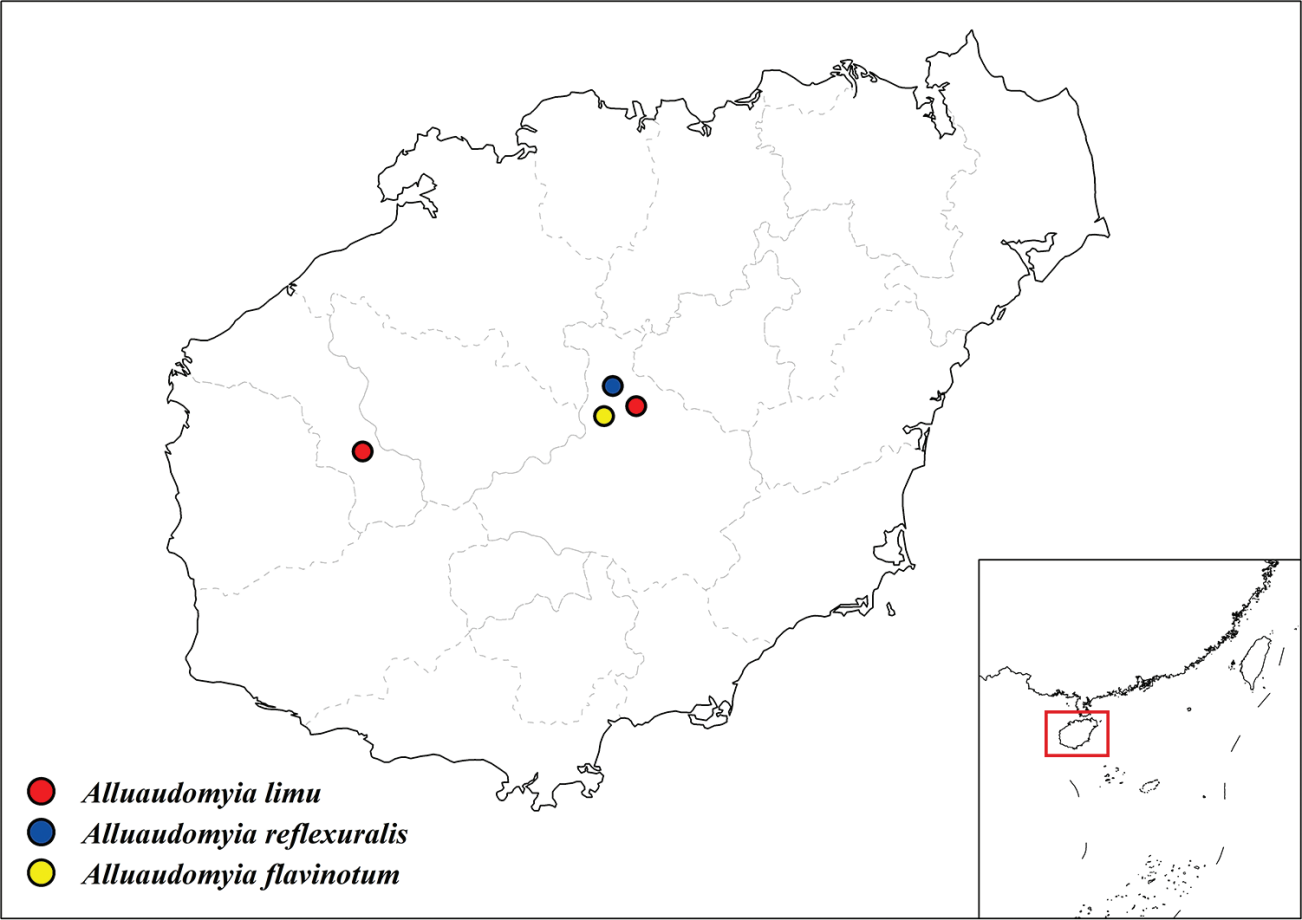
Geographical distribution of the three new species on Hainan Island. Hainan Island is also shown in the inset in the context of southern China.

China: Hainan Island: Qiongzhong and Changjiang County (Fig. 9).

##### Remarks.

All specimens of *A.limu* were collected from the rainforest of Hainan Island at altitudes of 567–817 m. Males and females were associated based on DNA barcodes.

### ﻿COI barcodes divergence and taxon identification tree

Three new species were identified based on morphological characters, and DNA barcoding was conducted for further identification. Twenty-two partial sequences of 638–658 bp of COI were successfully obtained from the three new species (GenBank accession numbers: *A.flavinotum*: OM722201–OM722203, *A.reflexuralis*: OM722204–OM722216, and *A.limu*: OM722217–OM722222) in this study, including three females of *A.flavinotum*, eight females and five males of *A.reflexuralis*, and five females and one male of *A.limu*.

Intra- and interspecific genetic distances based on COI were calculated for species of the genus *Alluaudomyia*. The intraspecific genetic divergence was low for *Alluaudomyia* species: *A.flavinotum* (0.004–0.005, *n* = 3), *A.reflexuralis* (0.000–0.017, *n* = 13), *A.limu* (0.000–0.004, *n* = 6) and *A.parva* (0.004–0.032, *n* = 9). While the interspecific genetic divergence between the five *Alluaudomyia* species ranged from 0.114–0.193.

**Figure 10.**
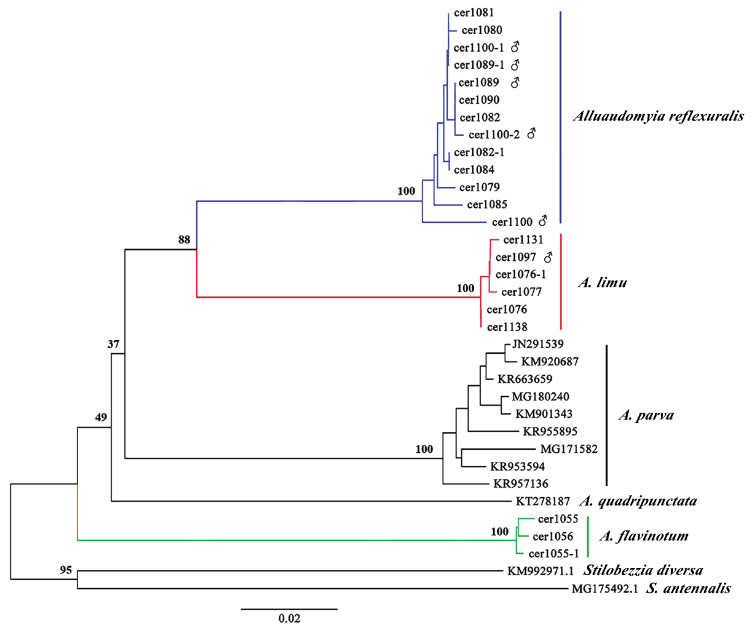
NJ tree for species of the genus *Alluaudomyia* inferred from mtDNA COI region. Bootstrap support values are displayed upper the nodes.

Thirty-two sequences of *Alluaudomyia* species and two sequences of *Stilobezzia* species were used to reconstruct NJ trees. The dendrogram of the NJ tree (Fig. 10) shows five clades, each representing a single species and supporting the morphological identifications.

## ﻿Discussion

Within the genus *Alluaudomyia* the species are more or less pigmented on their wings, legs, and remainder of the body (Borkent 2017). Pigmentation patterns are usually good characters for identifying species and associating sexes. The opposite sex is usually associated based on localities and the pigmentation patterns when a new *Alluaudomyia* species is proposed, but it is always challenging to correctly associate females with males if they lack distinguishing patterns of pigmentation. DNA barcoding was used here to associate males and females by providing independent evidence of their species’ status. DNA barcodes are also useful in both identifying the new species described herein and clarifying the status of other species of the genus *Alluaudomyia*.

## Supplementary Material

XML Treatment for Alluaudomyia flavinotum

XML Treatment for Alluaudomyia reflexuralis

XML Treatment for Alluaudomyia limu
